# Smartphone-Based VO2max Measurement With Heart Snapshot in Clinical and Real-world Settings With a Diverse Population: Validation Study

**DOI:** 10.2196/26006

**Published:** 2021-06-04

**Authors:** Dan E Webster, Meghasyam Tummalacherla, Michael Higgins, David Wing, Euan Ashley, Valerie E Kelly, Michael V McConnell, Evan D Muse, Jeffrey E Olgin, Lara M Mangravite, Job Godino, Michael R Kellen, Larsson Omberg

**Affiliations:** 1 Sage Bionetworks Seattle, WA United States; 2 Exercise and Physical Activity Resource Center University of California at San Diego San Diego, CA United States; 3 Department of Genetics Stanford University School of Medicine Stanford, CA United States; 4 Department of Rehabilitation Medicine University of Washington Seattle, WA United States; 5 Stanford University School of Medicine Stanford, CA United States; 6 Google Health Palo Alto, CA United States; 7 Scripps Research Translational Institute and Scripps Clinic La Jolla, CA United States; 8 Division of Cardiology and the Cardiovascular Research Institute University of California San Francisco San Francisco, CA United States

**Keywords:** VO2max, heart rate, digital health, real-world data, cardiorespiratory fitness, remote monitoring, mobile phone, smartphone, validation

## Abstract

**Background:**

Maximal oxygen consumption (VO_2_max) is one of the most predictive biometrics for cardiovascular health and overall mortality. However, VO_2_max is rarely measured in large-scale research studies or routine clinical care because of the high cost, participant burden, and requirement for specialized equipment and staff.

**Objective:**

To overcome the limitations of clinical VO_2_max measurement, we aim to develop a digital VO_2_max estimation protocol that can be self-administered remotely using only the sensors within a smartphone. We also aim to validate this measure within a broadly representative population across a spectrum of smartphone devices.

**Methods:**

Two smartphone-based VO_2_max estimation protocols were developed: a 12-minute run test (12-MRT) based on distance measured by GPS and a 3-minute step test (3-MST) based on heart rate recovery measured by a camera. In a 101-person cohort, balanced across age deciles and sex, participants completed a gold standard treadmill-based VO_2_max measurement, two silver standard clinical protocols, and the smartphone-based 12-MRT and 3-MST protocols in the clinic and at home. In a separate 120-participant cohort, the video-based heart rate measurement underlying the 3-MST was measured for accuracy in individuals across the spectrum skin tones while using 8 different smartphones ranging in cost from US $99 to US $999.

**Results:**

When compared with gold standard VO_2_max testing, Lin concordance was *p*_c_=0.66 for 12-MRT and *p*_c_=0.61 for 3-MST. However, in remote settings, the 12-MRT was significantly less concordant with the gold standard (*p*_c_=0.25) compared with the 3-MST (*p*_c_=0.61), although both had high test-retest reliability (12-MRT intraclass correlation coefficient=0.88; 3-MST intraclass correlation coefficient=0.86). On the basis of the finding that 3-MST concordance was generalizable to remote settings whereas 12-MRT was not, the video-based heart rate measure within the 3-MST was selected for further investigation. Heart rate measurements in any of the combinations of the six Fitzpatrick skin tones and 8 smartphones resulted in a concordance of *p*_c_≥0.81. Performance did not correlate with device cost, with all phones selling under US $200 performing better than *p*_c_>0.92.

**Conclusions:**

These findings demonstrate the importance of validating mobile health measures in the real world across a diverse cohort and spectrum of hardware. The 3-MST protocol, termed as *heart snapshot,* measured VO_2_max with similar accuracy to supervised in-clinic tests such as the Tecumseh (*p*_c_=0.94) protocol, while also generalizing to remote and unsupervised measurements. *Heart snapshot* measurements demonstrated fidelity across demographic variation in age and sex, across diverse skin pigmentation, and between various iOS and Android phone configurations. This software is freely available for all validation data and analysis code.

## Introduction

### Background

Expanding access to precision medicine will increasingly require patient biometrics to be measured in remote care settings. Traditionally, cardiovascular health has been assessed using risk scores such as the Framingham Risk Score [1], Reynolds Risk Score [2], Qrisk [3], and others, which integrate multiple factors including demographic data, comorbidities, and biometrics paired with imaging-based assessments measuring vascular blockage and blood flow in higher-risk and symptomatic individuals. Although these factors have a clear correlation with cardiovascular health, their inclusion in integrative risk calculations was promoted in part because they can be rapidly evaluated across many individuals. However, one of the most predictive biometrics for cardiovascular health [4] and overall mortality [5], maximal oxygen consumption (VO_2_max), is typically not incorporated in these risk calculators because of the high cost, participant burden, and specialized equipment and staff needed to obtain this measurement [6,7].

Cardiorespiratory fitness, as measured by VO_2_max, represents the integrated function of physiological systems involved in transporting oxygen from the atmosphere to the skeletal muscles to perform physical work. Existing gold standard techniques for measuring VO_2_max are based on protocols that use exercise on a treadmill or stationary bicycle paired with the direct measurement of oxygen consumption at various workloads, including maximal exertion [8,9]. However, the requirement to exercise at the maximal aerobic threshold limits deployment in some populations for safety reasons, and the need for specialized equipment and personnel has prohibited widespread adoption of VO_2_max testing in research and clinical settings.

### Objectives

Limitations of gold standard VO_2_max measurements have led to the development of numerous ”silver standard” [10] VO_2_max estimation protocols that rely on simpler equipment or submaximal levels of exertion. These protocols trade off measurement accuracy for ease of deployment in a wider range of settings and for populations with differing levels of capacity [11]. However, these protocols were typically developed and validated in small, homogeneous populations, and some subsequent validation studies have been criticized for demonstrating participant selection bias [12]. To overcome these limitations, we aim to develop a digital VO_2_max estimation protocol that could be self-administered remotely using only the sensors within a smartphone, and we also aim to validate this measure within a broadly representative population. Previous efforts have used a smartphone-based approach to measure VO_2_max, but these validation studies are rarely conducted outside of clinical settings [13]. Therefore, we aim to validate our measurements in remote, unsupervised real-world settings.

## Methods

### Development of Smartphone Sensor–Based Measurements of VO_2_max

Two silver standard VO_2_max estimation protocols were chosen as the basis for developing the smartphone tests. The first is the Cooper protocol [14], comprising a 12-minute run test (12-MRT), where individuals cover as much distance as possible in 12 minutes on a flat course. The Cooper protocol estimates VO_2_max from the total distance traveled during the 12 minutes. The other is the Tecumseh protocol [15], which comprises a 3-minute step test (3-MST), where individuals step up and down an 8-inch step at a constant rate for 3 minutes. In the 3-MST protocol, VO_2_max was estimated from the heart rate measurements during the recovery period. In adopting these protocols for smartphones, we developed self-guided instructions with GPS to record distance during the 12-MRT and a smartphone camera to record heart rate during recovery for the 3-MST (Multimedia Appendix 1).

### VO_2_max Validation Cohort Procedures and Measures

All study procedures were approved by the University of California, San Diego (UCSD) Institutional Review Board (approval number 171815). All participants provided written informed consent and attended two in-person study visits at the Exercise and Physical Activity Resource Center (EPARC).

A convenience sample of 101 adults aged between 20 and 79 years was recruited, largely balanced across age deciles and sex (Multimedia Appendix 2). Potential participants were contacted by trained EPARC staff via email or telephone and they underwent a screening to ascertain their eligibility. Participants were included if they were (1) able to consent and participate in the study using English; (2) aged between 20 and 79 years; (3) willing and able to attend two in-person study visits that included either a VO_2_max test or a 12-MRT and a 3-MST within a 2-week period; (4) willing and able to undertake up to three 12-MRT and 3-MST at home over a 2-week period; (5) willing and able to download the smartphone app developed to measure cardiorespiratory fitness on a compatible Android or iOS device and use it during all tests over a 2-week period; and (6) willing and able to download the Fitbit smartphone app on a compatible Android or iOS device and connect and wear a study-provided Fitbit Charge 2 during all tests over a 2-week period. Participants were excluded if they (1) were >12 weeks pregnant; (2) had a heart or cardiovascular condition, including coronary artery disease, congestive heart failure, diagnosed abnormality of heart rhythm, atrial fibrillation, and/or a history of myocardial infarction; (3) required the use of an external device to assist heart rhythm (eg, a pacemaker); (4) had a serious respiratory disease, including chronic obstructive pulmonary disease, exercise-induced asthma, and/or pulmonary high blood pressure; (5) required use of supplemental oxygen; (6) required use of a beta-blocker or other medications known to alter heart rate; and (7) answered “yes” to one or more questions in the American College of Sports Medicine’s Physical Activity Readiness Questionnaire and/or reported two or more risk factors for exercise testing and did not receive subsequent medical clearance. The Physical Activity Readiness Questionnaire is a widely accepted tool used to assess an individual’s fitness for tests involving cardiovascular exercise [16].

Upon completion of the telephone screening (and, if necessary, receipt of medical clearance), potential participants were scheduled to attend the first testing session at the UCSD. They were asked to report to the testing session well hydrated and in an athletic attire. Participants were guided through the process of downloading and installing the smartphone app developed to measure cardiorespiratory fitness, as well as the Fitbit smartphone app, and they were fitted with a wrist-worn Fitbit Charge 2 according to the manufacturer’s recommendations. Participants were asked to provide their age, sex at birth, ethnicity, and race. Weight (to the nearest 0.1 kg) and height (to the nearest 0.1 cm) were measured using a calibrated digital scale and stadiometer (Seca 703, Seca GmbH & Co. KG). Both weight and height were measured with participants wearing lightweight clothes without shoes, and two separate measurements were averaged (if weight or height measurements differed by more than 1%, then a third measure was taken, and the average of the two measures that differed by less than 0.2 kg or 0.5 cm, respectively, was used).

At the first testing session, participants either undertook a VO_2_max test or an in-clinic 3-MST and 12-MRT. A randomization procedure implemented before the scheduling of the first testing session determined which test procedure participants undertook during the first testing session. The participants were then expected to complete the other test procedures during the second testing session.

### Treadmill-Based Gold Standard VO_2_max Measurement

Participants completed a maximal graded exercise test on a Woodway 4Front treadmill (Woodway) calibrated monthly for accuracy of speed and grade. The maximal graded exercise test protocol began with a warm-up at a self-selected pace on a treadmill for 5 to 10 minutes. During the warm-up, EPARC staff explained how to use the Borg Rating of Perceived Exertion scale and reminded participants that they were expected to achieve their maximal level of exertion [17].

The participants were then equipped with a breath mask that covered the nose and mouth (KORR Medical Technologies) and a Bluetooth-enabled heart rate monitor worn on the chest (Garmin). The preprogrammed treadmill protocol began with the participants running at 5 mph with a 0% incline for 3 minutes. The workload was then increased by approximately 0.75, which is the metabolic equivalent of tasks every minute. This was achieved via an increase in speed (0.5 mph per min) each minute until the participant was 0.5 mph above their self-determined comfortable speed or until a maximal speed of 9 mph was reached. If the participant’s capacity allowed them to continue beyond this upper speed limit before reaching volitional fatigue, then the treadmill speed was kept constant, but the grade (ie, incline) of the treadmill was increased by 1% each minute until volitional fatigue was reached. The Borg Rating of Perceived Exertion scale was assessed during the final 10 seconds of each minute, and the protocol continued until the participant signaled to stop (ie, indication of volitional fatigue). Upon indication of volitional fatigue, the treadmill was immediately slowed to 2 mph, and participants were encouraged to walk until completely recovered. Breath-by-breath oxygen uptake (VO_2_) was continuously measured using an indirect calorimeter (COSMED) that was calibrated for gas volume and fractional composition immediately (ie, <30 min) before the start of the maximal graded exercise test protocol.

### Tecumseh Test (3-MST) and Cooper Test (12-MRT) In-Clinic Procedure

All participants were fitted with a chest-worn heart rate monitor (Polar) that was used for real-time monitoring by trained EPARC staff throughout both the 12-MRT and 3-MST. For the 3-MST, participants were instructed to step up and down from a single step 8 inches in height at a rate of 24 steps per minute for 3 minutes [18]. The cadence of stepping was monitored by trained EPARC staff. The radial pulse was measured from the 31st second to the 60th second after 3 minutes of stepping. Upon completion of the test, the participants were asked to sit in a chair and rest. After a minimum of 10 minutes of rest, participants completed a 5-minute self-determined light intensity warm-up. They were then instructed to cover as much distance as possible on a flat 400-m track for 12 minutes. The distance traveled was measured after 12 minutes [14].

### Distance Estimation Using Privacy-Preserving GPS Data

The distance recorded by the smartphone during the 12-MRT was validated against the actual distance. The smartphone recorded displacement information sampled at 1 Hz, which consists of relative location measurements, that is, the change in location with regard to the last recorded measurement. The iPhones (Apple Inc) measured displacement in meters whereas the Android smartphones measured relative changes in latitude and longitude, requiring an estimate of the absolute latitude and longitude to be added back into the measurements to obtain an accurate estimate of distance.

The first distance estimation method entailed summing the Euclidean distances between subsequent GPS points. As GPS measurements have a range error dependent on atmospheric effects and numerical errors, a second method was used to compute the distance after smoothing the trajectory of the GPS path using a Savitzky-Golay smoothing filter.

### Camera-Based Heart Rate Estimation

Blood flow through the fingertip was measured through video with a rear-facing camera while the flash was on. The resting heart rate was captured with 20 seconds of recording, whereas the 3-MST required 60 seconds of recording. During the capture, we found it was important to fix the focal length to infinity, turn off any high dynamic range settings (if applicable), and set the frame rate to 60 Hz if possible, and if not, the default highest allowed by the phone. We did not record the video in order to preserve privacy associated with the inadvertent capture of identifiable objects in the frame before covering the lens with the finger, but instead summarized each video frame to the mean of all pixel intensities per color channel in the red, green, blue space.

These intensities yielded three time series, one for each color. These time series were filtered and mean-centered before being split into shorter 10-second windows. By assuming a periodic signal for these windows, the autocorrelation function (ACF) was used to estimate the period by finding the peaks and their corresponding lags. The relative magnitude of the peaks to the maxima of the ACF was used to generate a confidence score, which quantifies the extent to which the signal is periodic or if the peak at the fundamental frequency (ie, the peak with the highest magnitude) is a spurious peak. The ACF is calculated over a 10-second window, as this provides sufficient heart beat observations postprocessing to estimate heart rates ranging from 45 to 210 bpm.

To filter potentially spurious peaks, a magnitude threshold relative to the magnitude of the peak at zero lag was used. The confidence score was calculated as the ratio of the magnitude of the peak corresponding to the fundamental frequency to the next peak. The confidence score is an indicator of the periodicity of the signal, a property indicative of the heart rate signal in a short finite time window. The different color channels were merged by choosing the heart rate estimate from the channel (red or green) that had the maximum confidence score within a given window.

### Estimation of VO_2_max

#### 3-Minute Step Test

Multiple formulas for predicting VO_2_max from the Tecumseh step test and its variations have been developed [15]; here, we used the following established by Milligan [19]:

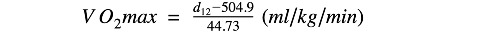



where HB3060 is the number of beats between *30* and *60* seconds after the step test, *age* is the age of the subject, and *sex* is *0* if male and *1* if female.

#### 12-Minute Run Test

VO_2_max for the 12-MRT is estimated from the following formula, where *d*_12_ is the distance covered in meters [14]:

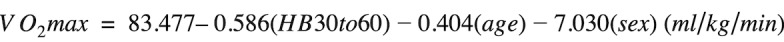



### Heart Rate Calibration Study Procedures and Measures

All study procedures were approved by the UCSD Institutional Review Board (approval number 181820). All participants provided written informed consent and attended one in-person study visit at the EPARC.

A convenience sample of 120 adults, aged 18-65 years, of six different skin types were asked to participate in this study. We aimed to recruit an equal ratio of male and female participants, as well as an equal number of participants with each skin type, as determined by the Fitzpatrick scale. Participants were included if they were (1) able to consent and participate in the study in English and (2) aged between 18 and 65 years. Participants were excluded if they had (1) peripheral neuropathy or (2) tattoos or scarring at the measurement site (index finger and/or wrist). Potential participants were contacted by trained EPARC staff via email or telephone, and they were asked to complete the screening to ascertain their eligibility.

To establish the Fitzpatrick skin type of the cohort during recruitment, participants were asked to self-assess their Fitzpatrick skin type based on visual comparison with images of well-known celebrities with diverse pigmentation levels. As self-assessment of skin type can have variable accuracy [20,21], spectrocolorimetry was also used as an objective standard [22]. Spectrocolorimetry measurements were performed on the underside of the jaw using Pantone RM200QC. To calculate pigmentation in the individual typology angle color space, the L^*^ and b^*^ parameters from the spectrocolorimetry measurements were used according to the formula:

individual typology angle = [arctan((L^*^−50)/b^*^)] × 180/3.14159 **(1)**

Using this formula, skin color types can be classified into six groups, ranging from very light to dark skin: very light>55°>light>41°>intermediate>28°>tan>10°>brown>−30°>dark [22].

Upon completion of the telephone screening, potential participants were scheduled to attend the first testing session at the UCSD. Participants were asked to provide their age, sex at birth, ethnicity, and race. All participants were fitted with a chest-worn heart rate monitor that was used for real-time monitoring by trained EPARC staff throughout testing. Heart rate was also monitored using a finger-based pulse oximeter (Nonin Medical, Inc). The finger-based pulse oximeter was attached to the participants’ index finger, and the time was synchronized between the computer and the device. Trained research staff visually confirmed that the photoplethysmograph was reading accurately before starting measurements on smartphone devices.

Participants were then given the first of 8 smartphones: Huawei Mate SE, LG Stylo 4, Moto G6 Play, Samsung Galaxy J7, Samsung Galaxy S9+, iPhone8+, iPhoneSE, and iPhoneXS. They were instructed by trained research staff to stand still and gently cover the camera and flash on the back of the smartphone with their fingertip, as their heart rate was captured by our preloaded smartphone app. The time on the Polar app was recorded at the time the measurement began on the smartphone app. Measurements with each smartphone lasted 60 seconds in duration. Processed data from the finger-based pulse oximeter were parsed and transformed with custom scripts to generate continuous photoplethysmography data in a format suitable for comparison with the heart rates from the phones.

### Statistical Analysis

Demographic data were described using univariate summary statistics (eg, proportions, means, and SDs). Test validity for heart rate estimates and VO_2_max was visualized using Bland-Altman plots [23] and compared using the Lin concordance index [24]. The heart rate errors were also compared using percent error. Analyses were performed in both R and Python.

### iOS and Android Heart Snapshot Software Modules

The code for the heart snapshot modules and sample Android [25] and iOS [26] apps are available under an open-source license.

## Results

### Validation in a Clinical Setting

**Figure 1:**
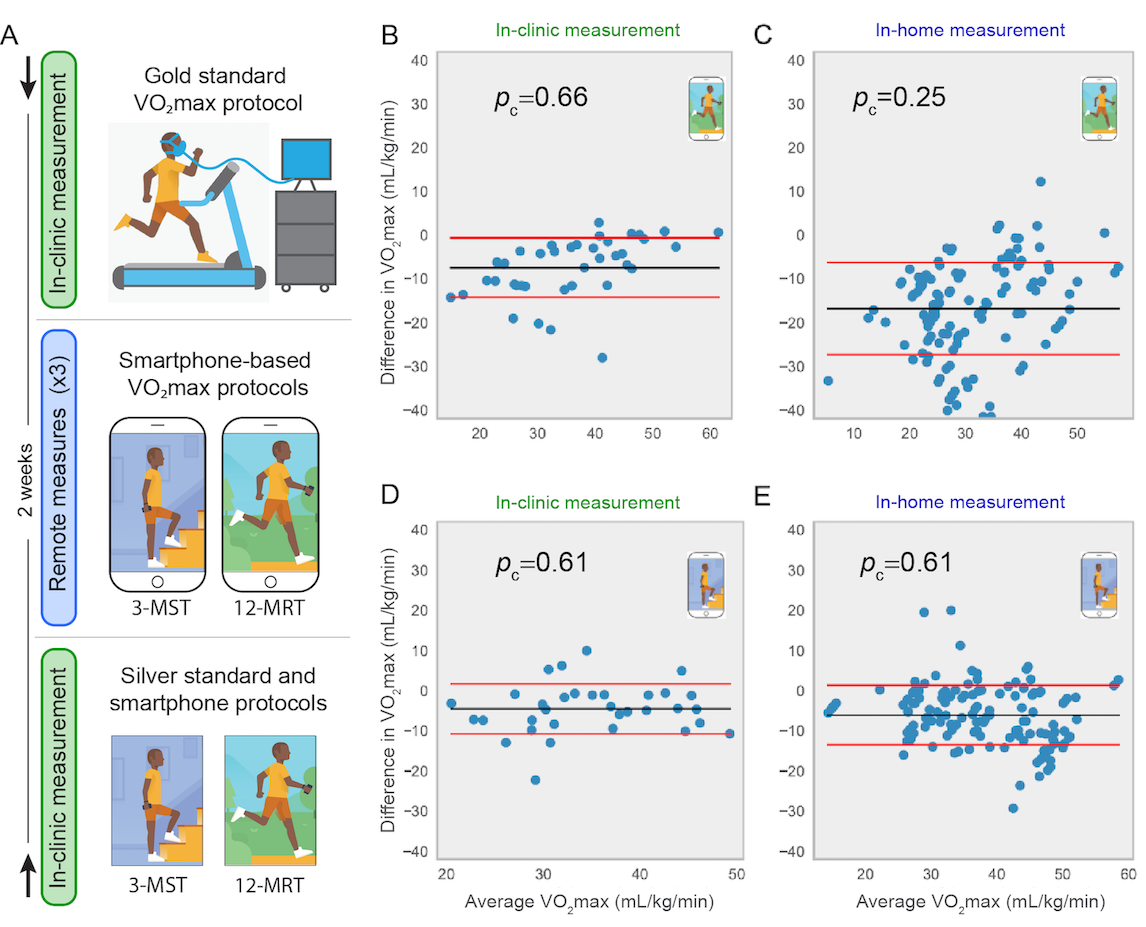
Validation protocol and primary results of validation. (A) Participants in the study were randomized into two groups. The first group (denoted by the downward-facing arrow at top) performed a gold standard VO_2_max protocol and received training on day 1. The second group performed the two silver standard protocols concurrently with the smartphone protocols on day 1 (denoted by the upward-facing arrow at bottom). Both groups then performed the two smartphone protocols remotely up to three times during a 2-week period. (B) to (E) show Bland-Altman plots comparing the gold standard VO_2_max with smartphone measures from: (B) 12-MRT performed in clinic, (C) 12-MRT performed remotely (up to 3 repeats per participant), (D) 3-MST in clinic, and (E) 3-MST remotely. VO_2_max: maximal oxygen consumption; 3-MST: 3-minute step test; 12-MRT: 12-minute run test.

To assess the validity of the 3-MST and 12-MRT smartphone measurements, gold standard VO_2_max treadmill testing was performed with 101 participants distributed across age deciles 20-80 years. Every participant also performed the silver standard and smartphone 12-MRT and 3-MST protocols in the clinic, with three instances of each smartphone protocol performed over 2 weeks without supervision in the participant’s home environment (Figure 1).

**Figure 2:**
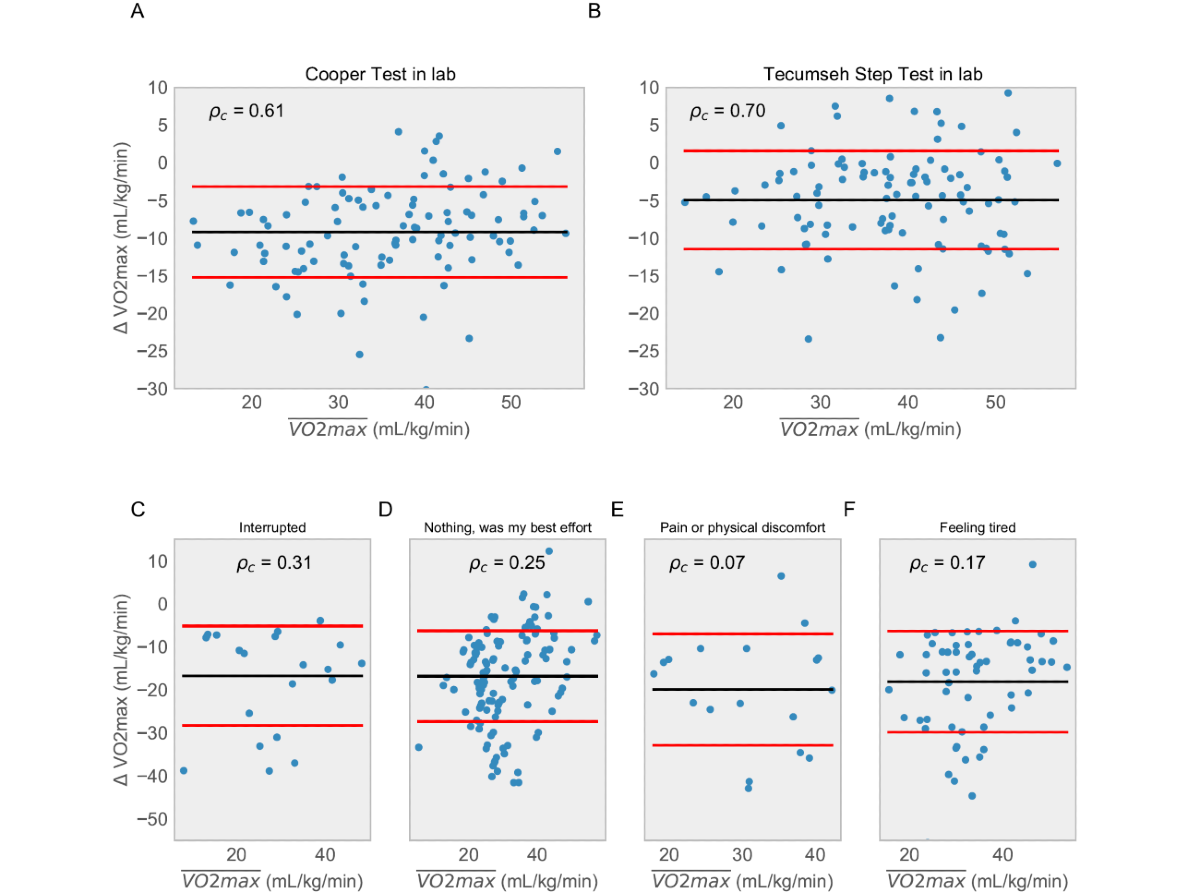
Comparison of in-clinic performance of silver standard protocols relative to the gold standard for (A) 12-minute run test (12-MRT) and (B) 3-minute step test. For each plot, we are showing the difference between the ground truth maximal oxygen consumption measurement and measurements obtained using the distance run around a track (for A) and heart rate via radial pulse measured by trained research staff (for B) as per Tecumseh protocol. This distance was also measured using GPS and heart rate was measured using a chest strap and Fitbit. The concordance between distance measured around the track and measured using the GPS in the phone was 0.96. (C) to (F) show the concordance of the 12-MRT test for different values of self-reported effort. VO_2_max: maximal oxygen consumption.

The in-clinic 12-MRT distance was measured on a 400-m track and by the smartphone GPS. The in-clinic heart rate was measured via radial pulse by trained research staff, a chest-worn Polar heart monitor, a wrist-worn Fitbit Charge 2, and a smartphone camera with the flash activated. Comparisons between the gold standard, silver standard, and smartphone-based protocols for VO_2_max estimation were performed using Bland-Altman analysis [23] and the Lin concordance index (*p*_c_). The concordance between gold standard VO_2_max and the silver standard Cooper protocol (*p*_c_=0.61; Figure 2) and the silver standard Tecumseh protocol (*p*_c_=0.70; Figure 2) were in line with previously published results [27-29]. Concordance of smartphone-based protocols with gold standard VO_2_max testing was *p*_c_=0.66 for the 12-MRT (Figure 1) and *p*_c_=0.61 for the 3-MST (Figure 1). The concordance of smartphone-based protocols with silver standard protocols was *p*_c_=0.96 for the 12-MRT and *p*_c_=0.94 for the 3-MST. These results demonstrate that the smartphone-based protocols fall short of recapitulating gold standard VO_2_max testing but are highly concordant with validated silver standard VO_2_max estimation protocols in a laboratory setting.

### Validation in a Remote Setting

To investigate whether the concordance of in-clinic measurements would generalize to remote and unsupervised settings, the smartphone protocols were also performed up to three times at home by each participant. We observed an approximately equal test-retest reliability between the two tests (3-MST intraclass correlation coefficient=0.86; 12-MRT intraclass correlation coefficient=0.88). However, although the 3-MST translated well to an unsupervised setting (*p*_c_=0.61; Figure 1), the 12-MRT demonstrated a pronounced drop in concordance (*p*_c_=0.25; Figure 1), despite a highly accurate distance measurement from the smartphone (*p*_c_=0.96) based on comparisons made in a clinical setting.

As the 12-MRT is dependent on maximal effort, participants were surveyed directly after their run about their performance. In 63.4% (137/216) of runs performed remotely, participants reported the run to be “their best effort.” Therefore, only 137 runs were used to estimate VO_2_max in our analysis. Figure 2 captures the results of all 216 runs subdivided by self-reported effort. Although the context-dependent failure of the 12-MRT in remote settings may be attributable to many factors, this result highlights the importance of both clinical and unsupervised real-world evidence for the validation of novel digital health measurement modalities.

### Calibration of the Heart Snapshot Measurement for a Diverse Audience

**Figure 3:**
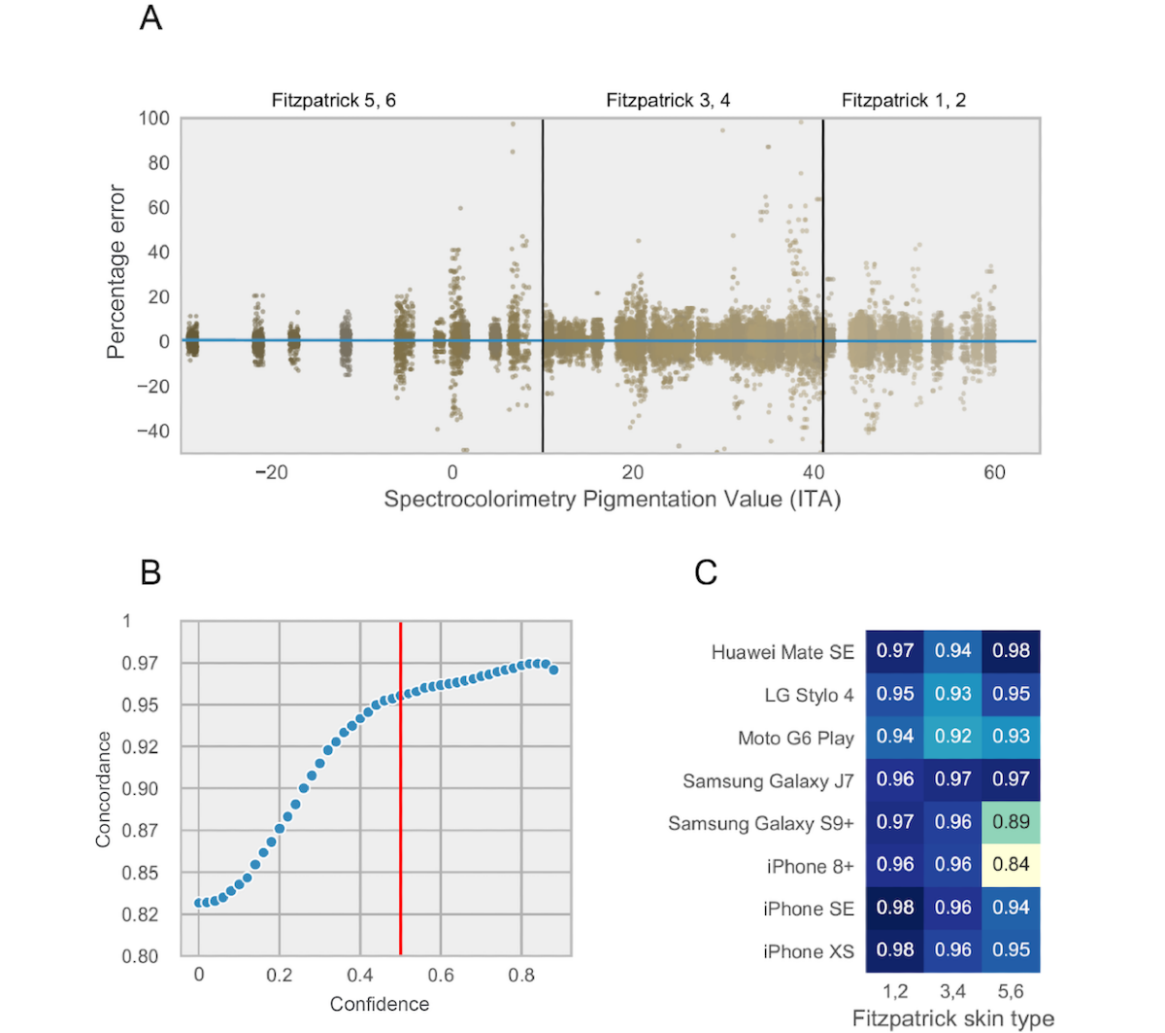
Validation of heart rate measurements across different skin tones and hardware configurations in the calibration study. (A) Percent error in heart rate estimation from ground truth as a function of different colors captured by spectrocolorimetry under the jaw. Each dot represents a 10 second window of heart rate in one individual. (B) Distribution of concordance between heart rate using pulse oximetry and smartphone as the confidence cutoff is changed. Red line represents the chosen cutoff used for analysis. (C) Concordance as a function of smartphone models and Fitzpatrick skin tones. ITA: individual typology angle.

The smartphone-based 3-MST protocol, hereafter referred to as *heart snapshot*, was generalizable between clinical and remote assessments and was robust over a large range of fitness levels. Maintaining high concordance during unsupervised measurements is necessary to achieve the scale intended for the targeted 1 million participants in the *All of Us* Research Program (AoURP) [30], which will use a “bring-your-own-device” strategy for remote self-measurement of VO_2_max. AoURP also aims to recruit a study population matching the full demographic diversity of the United States, emphasizing the inclusion of groups often underrepresented in biomedical research, such as ethnic and racial minorities. As prior studies have shown differing results as to whether optical techniques for heart rate detection (photoplethysmography) can be demographically biased [31,32], we aimed to investigate any differences in *heart snapshot* accuracy across variations in skin tone. A follow-up calibration study for heart rate measurements was conducted with 120 participants distributed approximately evenly across defined Fitzpatrick skin types [33], using 8 different smartphones (3 iPhones and 5 Android smartphones ranging in cost from US $99 to US $999 at the time of writing). These phones were chosen to be representative of different operating systems, quality of sensors, processing speed, and camera configuration. Importantly, we observed no significant difference in heart rate measurement accuracy between categorical Fitzpatrick skin types or systematic measurement error proportional to skin color at either end of the spectrum (Figure 3).

### Internal Quality Control Procedures for Heart Snapshot

**Figure 4:**
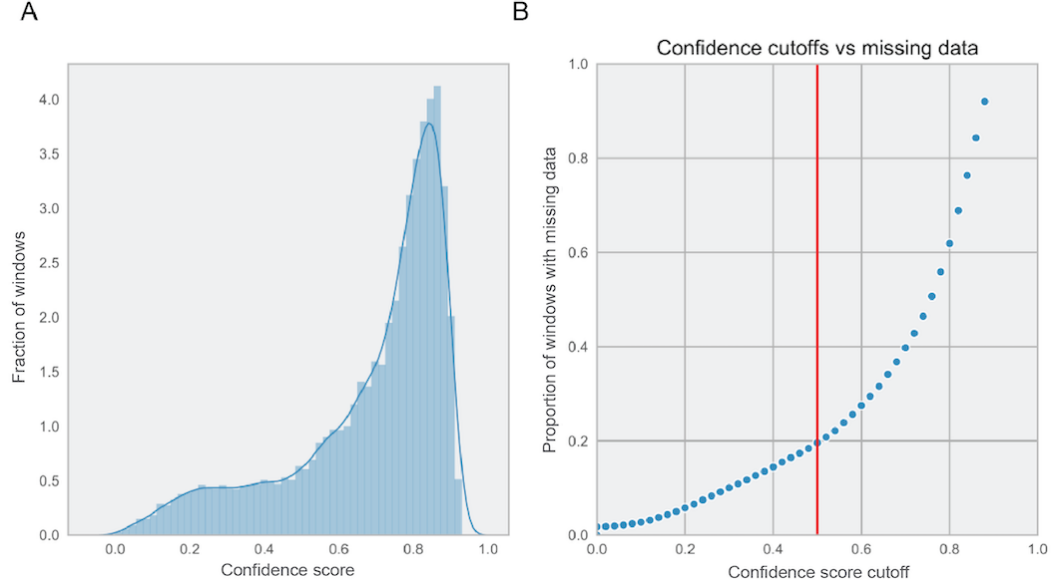
Effect of different confidence cutoff on the amount of missing data from the calibration study. (A) Distribution of best confidence across red and green channels in the calibrations study and (B) percent of the 10 second windows that are filtered out at different cutoffs of the confidence score. The cutoff used in the analysis is 0.5 marked by the red line.

**Figure 5:**
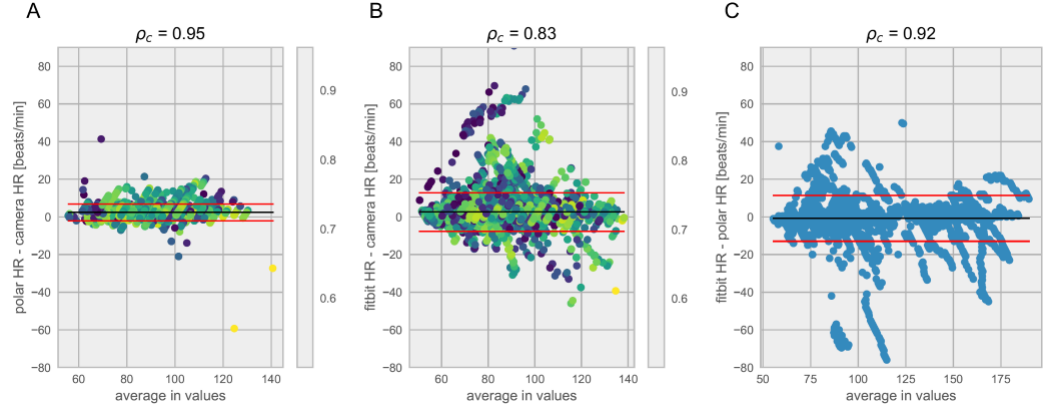
Bland-Altman analysis comparing heart rate measurements in the validation study using data collected during the Tecumseh tests. In the validation cohort, participants used multiple ways of collecting heart rate. The method being tested, the smartphone camera, was compared to: (A) a Polar chest strap (considered a gold standard) while in the clinic when both were used and (B) a Fitbit worn during the entirety of the study. (C) We also compared the Polar strap to the Fitbit for all time that both were worn. HR: heart rate.

To facilitate quality control of the measurements across different smartphones, a confidence score was developed to provide a readout of the quality of the heart rate measurements. This confidence score is derived from the ACF of the heart rate signal across 10-second measurement windows. Using the calibration study results, a balance between the quality of measurements was weighed against the loss of data by choosing a filtration cutoff at a confidence level of ≥0.5. This resulted in a *p*_c_=0.95, in the calibration cohort between a pulse oximetry pulse measurement and the camera-estimated heart rate (Figure 3). In selecting this confidence score as a cutoff, we observed that 80.41% (28,032/34,859) of all measurement windows were retained in this calibration cohort (Figure 4). The same cutoff was used in the validation of *heart snapshot* against gold standard VO_2_max, where the heart rate concordance with a chest-worn Polar heart monitor was *p*_c_=0.95 and *p*_c_=0.83 when compared with a wrist-worn Fitbit Charge 2, both at home and in the clinic. This can be compared with *p*_c_=0.92 between Polar and Fitbit Charge 2 (Figure 5).

Taken together, *heart snapshot* heart rate measurements in any of the combinations of the Fitzpatrick skin tones and 8 smartphones used in the calibration study resulted in a concordance greater than or equal to *p*_c_=0.84 (Figure 3), which is in line with previous smartphone-based modalities for heart rate monitoring [34]. Importantly, performance did not correlate with device cost, with all phones selling for under US $200 performing better than *p*_c_>0.92 for any skin tone.

## Discussion

### Principal Findings

In summary, *heart snapshot* measured VO_2_max with similar accuracy to supervised, in-clinic tests such as the Tecumseh or Cooper protocols, while also generalizing to remote and unsupervised measurements. *Heart snapshot* measurements demonstrated fidelity across demographic variation in age and sex, across diverse skin pigmentation, and between various iOS and Android phone configurations.

The results from our validation study performed in unsupervised, remote environments showed that heart snapshot, which is based on a 3-MST protocol, generalized to real-world settings but the 12-MRT protocol did not. Although it is difficult to definitively determine the reason for the poor concordance of 12-MRT, we suspect that this might be attributed to the Hawthorne effect, where people perform better when they are under constant observation at a track. It could also be purely environmental, where traffic, hills, and distractions impede uninterrupted running. This indicates the importance of testing and validating digital health measures in a representative setting.

### Limitations

An important limitation of this study is that we did not include any individuals in our cohort with a known irregular heart rhythm, so we cannot extend our claims of validity to that population. Similarly, although we made efforts to include individuals across different age deciles, we focused solely on adults (aged over 18 years) and our age decile from 60 to 70 years did not include participants older than 65 years. This work was limited to the biometrics of resting heart rate and VO_2_max, but using the same technology could also be extended to measure heart rate recovery in minute intervals after exertion, which would provide a valuable biometric that has been associated with prediction of overall mortality [30]. *Heart snapshot* attempts to maximize concordance with gold standard methods for estimating VO_2_max, but it is worth noting that this analysis used an existing validated algorithm [27] that was based on in-clinic procedures and measurement tools. *Heart snapshot* could become more personalized than traditional protocols, for example, adapting to a participant’s maximum step cadence as measured by smartphone accelerometry. Further concordance with gold standard measures may be achieved by optimizing the parameters of the traditional algorithm or including new variables, but this will require a distinct cohort to test any models that have been trained on this data set.

### Comparison With Prior Work

Although multiple devices can estimate VO_2_max, including several currently marketed consumer devices [35], the underlying data and algorithms are usually not published. The lack of data and method transparency limits the utility of these approaches for discovery-based research, where reproducibility is paramount. In contrast, an open approach to method validation can also serve as a foundation for downstream research in different conditions or populations to generate normative data for interpreting results [36].

As many dedicated hardware devices for digital health in the consumer sphere have experienced short half-lives of availability, we believe that the dependency only on a smartphone with a flash and camera may provide a greater degree of *future-proofing* for *heart snapshot*. This will be important for consistent, longitudinal measurements that may uncover patterns of VO_2_max variance over time, especially in large-scale studies such as the AoURP.

### Conclusions

The emerging development of consumer technology provides unprecedented opportunities to evaluate the use of additional digital biomarkers to improve risk management strategies for population health and for precision health at the level of an individual. Paired with access to large population studies, such as the AoURP [30] that collects health questionnaires, electronic health records, physical measurements, biospecimens, and digital health technology data, we can rapidly test emerging digital health measures for their potential to advance precision medicine. The *heart snapshot* software is freely available with all validation data and analysis code [37].

## Appendix

Multimedia Appendix 1 Self-guided instructions and screen workflow for performing the heart snapshot VO<sub>2</sub>max estimate.

Multimedia Appendix 2 Demographic data for the maximal oxygen consumption validation study.

## Abbreviations

3-MST: 3-minute step test
12-MRT: 12-minute run test
ACF: autocorrelation function
AoURP: All of Us Research Program
EPARC: Exercise and Physical Activity Resource Center
UCSD: University of California, San Diego
VO_2_max: maximal oxygen consumption
