# Supplementary figures and images for: Smartphone-Based VO2max Measurement With Heart Snapshot in Clinical and Real-world Settings With a Diverse Population: Validation Study
Source: JMIR Mhealth Uhealth. 2021 Jun 4;9(6):e26006. doi: 10.2196/26006 (PMC8214186; doi:10.2196/26006)

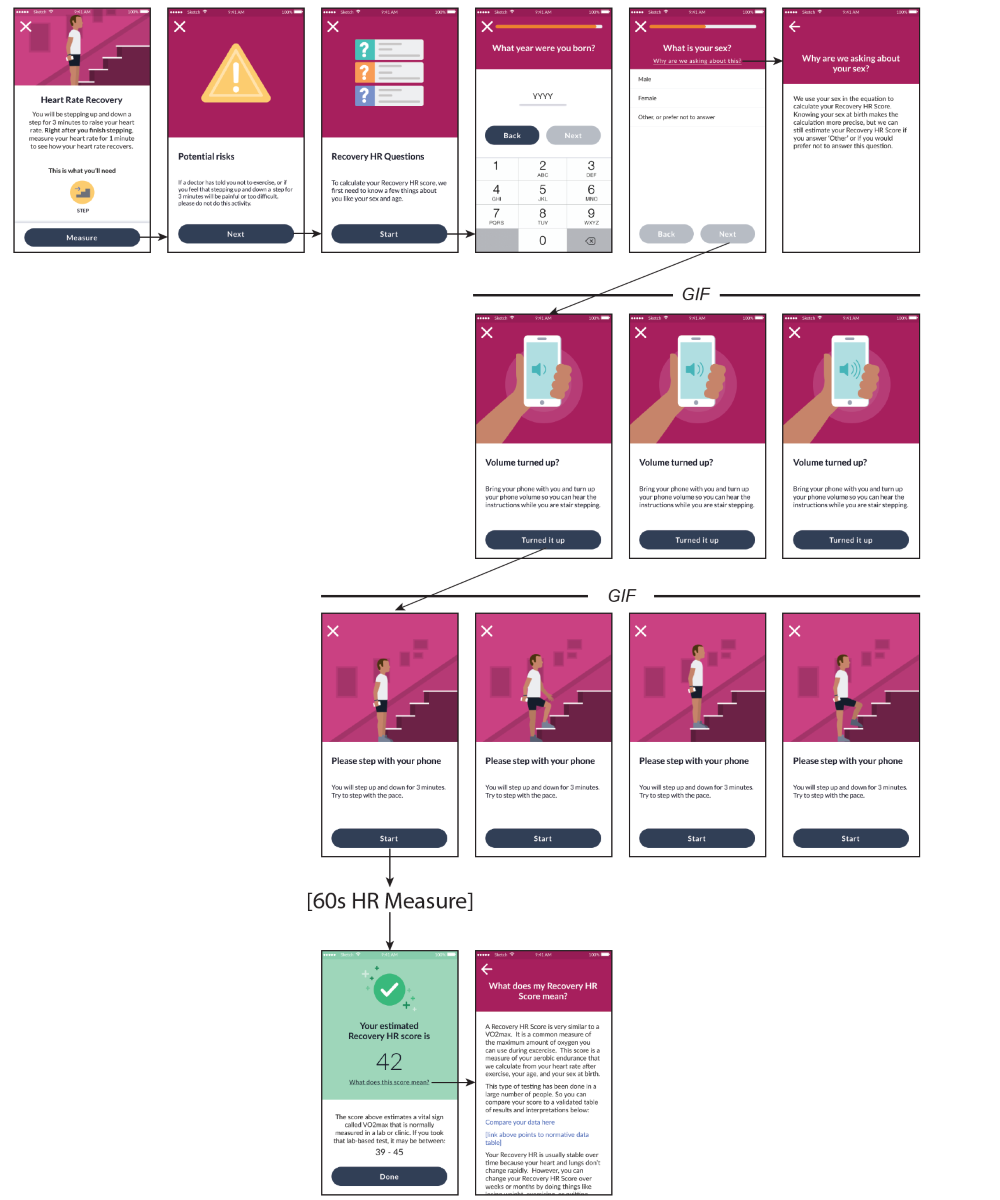

Supplement: Multimedia Appendix 1 [file mhealth_v9i6e26006_app1.png]
